# Polyphenolic inhibition of enterocytic starch digestion enzymes and glucose transporters for managing type 2 diabetes may be reduced in food systems

**DOI:** 10.1016/j.heliyon.2021.e06245

**Published:** 2021-02-12

**Authors:** Emmanuel O. Ayua, Smith G. Nkhata, Sydney J. Namaumbo, Elijah Heka Kamau, Theresa N. Ngoma, Kevin Omondi Aduol

**Affiliations:** a Department of Food Science and Nutrition, University of Eldoret, P.O Box 1125-30100, Eldoret, Kenya; b Agrofood Processing Technology, Faculty of Life Sciences and Natural Resources, Natural Resources College, Lilongwe University of Agriculture and Natural Resources, P. O Box 143, Lilongwe, Malawi; c Food Technology, Faculty of Life Sciences and Natural Resources, Natural Resources College, Lilongwe University of Agriculture and Natural Resources, P. O Box 143, Lilongwe, Malawi

**Keywords:** Polyphenols, Type II diabetes, Enzyme inhibition, Starch digestion, Glucose transporter

## Abstract

With the current global surge in diabetes cases, there is a growing interest in slowing and managing diabetes and its effects. While there are medications that can be used, they have adverse side effects such as hypoglycemia and weight gain. To overcome these problems, bioactive compounds commonly found in fruits, vegetables and cereal grains are used to slow starch digestion and transport of simple sugars across the intestinal epithelia thereby reducing plasma blood glucose spike. These effects are achieved through inhibition of amylases, glucosidases and glucose transporters present in the gastrointestinal tract and brush boarder membrane. The extent of inhibition by polyphenols is dependent on molecular structure, doses and food matrix. Glycemic lowering effect of polyphenols have been demonstrated both in i*n vivo* and *in vitro* studies. However, when these compounds are incorporated in food systems, they can interact with other polymers in the food matrix leading to lesser inhibition of digestion and/or glucose transporters compared to isolated or pure compounds as often witnessed in most *in vitro* studies.

## 1 Introduction

Diabetes is a metabolic disorder characterized by chronic hyperglycemia resulting from either insufficient insulin secretion (Type I diabetes mellitus) or insulin resistance (Type II diabetes mellitus) with the latter being more prevalent (Kaku, 2010). Type II diabetes is characterized by impaired metabolism of carbohydrates, lipids and proteins (Dasgupta and Klein, 2014) largely influenced by a combination of genetic and environmental factors such as sedentary lifestyle, stress, aging and increased calorie intake (Kaku, 2010; Palicka, 2002). Generally, there has been a global increase in diabetes cases in the past decades from 108 to 422 million between 1980 to the year 2014 with a higher incidence in new cases being observed in low and middle income nations than higher income ones (WHO, 2020) thereby calling for a need to address the condition.

To manage these cases, both medical and nutritional approaches are currently being used. Medical approach has employed the use of drugs such as metformin, meglitinides, thiazolidinediones, dipeptidyl peptidase-4 inhibitors, glucagon like peptide-1 receptors agonists, acarbose, voglibose and miglitol (Wang et al., 2019; Maideen, 2019) while nutritional approach has focused on recommending diets that produce low postprandial blood glucose. Unfortunately, most of these drugs have undesirable effects such as flatulence, diarrhea and stomach distention, hypoglycemia, weight gain and are not recommended for people with liver diseases (Schernthaner et al., 2014; He et al., 2015; Li et al., 2019). As a result, exploring the potential of bioactive compounds such as polyphenols, known to moderate postprandial blood glucose, in management of diabetes seems viable as they appear to have no or less adverse side effects (Gothai et al., 2016). This is supported by a plethora of information showing an inverse association between polyphenol intake and Type II diabetes or cardiovascular diseases (Psaltopoulou et al., 2011; Santangelo et al., 2016; Lin et al., 2016).

Polyphenols are primarily obtained from cereals, fruits, vegetables and beverages such as tea, coffee and wine and include anthocyanins from berry fruits, flavanones from citrus fruits, flavonols from apples and phenolic acids from cereals (Bordenave et al., 2014; Rasouli et al., 2017; Li et al., 2017). Research advances have indicated that polyphenols can inhibit either starch hydrolyzing enzymes in the mouth and small intestines or glucose transporter residing on brush border membranes thereby reducing the amount of glucose in blood circulation (Johnston et al., 2002; Moser et al., 2018). Inhibition of starch digestion enzymes has sparked great interest in managing postprandial blood glucose as prolonged elevated blood glucose is characteristic or predisposing factor to diabetes. The main enzymes inhibited by phenolic compounds are α-amylase and glucosidases and the inhibition capacity is dependent on structure of the compound as well as dose (Lo Piparo et al., 2008; Liu et al., 2017). The concentration of glucose in blood system therefore depends on efficiency of starch hydrolysis and subsequent transport of the digestion products into the blood streams. Therefore, it sounds reasonable that slowing these processes can consequently be beneficial in managing type II diabetes. The other protective effects of polyphenols are due to their antioxidant properties, chelation of metal ions and increasing sensitivity of insulin receptors (Barrett et al., 2018; Bordenave et al., 2014; Moser et al., 2018).

Largely, most of these studies involved the use extracts or pure forms of polyphenols (Oboh et al., 2015; Johnston et al., 2002; Lo Piparo et al., 2008) and less in foods with phenolic compounds incorporated in them (Coe and Ryan, 2016; Sui et al., 2016a; Jun et al., 2014). Developing novel foods incorporated with phenolic compounds could help provide an insight into the health benefits of these compounds in food systems as there is a growing interest in understanding how phenolic compounds would be affected when incorporated in solid vs liquid foods. Therefore, this review seeks to provide a comprehensive understanding of current literature on the use of polyphenols for inhibition of starch digestion enzymes and glucose transporters in novel food systems.

## 2 Starch digestion and glucose transport

Starch is an important component in human diet contributing 60–70% of the total energy derived from cereals and pulses (Ao et al., 2012). Chemically, starch is a homopolysaccharide which consists of 80–85% branched chain amylopectin with α[1→4] and α[1→6] glycosidic linkages, and 15–20% linear chain amylose with α[1→4] glycosidic linkages (Srichuwong et al., 2005; Yoo and Jane., 2002; Esmaeili and Noorolahi, 2017; Giuberti et al., 2020). Starch is further classified as waxy or not depending on their amylose content. Waxy starches contain a minimal amount of amylose (<15%) in their granule composition. Normal starches have high content of amylose (20–35%) while hylon starches (high amylose) consist of more than 50% of amylose (Hung et al., 2007; Van der Maarel and Leemhuis, 2013). It has been suggested that, waxy starches requires higher energy for gelatinization probably due to its high crystallinity caused by higher level of amylopectin. Typically, starch with higher proportion of long chain amylopectin are digested slowly than those with shorter chains (Magallanes-Cruz et al., 2017). According to these authors, crystalline and amorphous starch regions are organized into concentric circles where channels and pores link the inner regions to surfaces. Consequently, starch degrading enzymes take longer to get through to the concentric rings in the crystalline granules to digest starch which partly explains the reasons crystalline starches have slow digestion property (Zhang et al., 2008; Magallanes-Cruz et al., 2017). Starch is also categorized based on digestibility as rapidly digestible starch (RDS), slowly digestible starch (SDS) and resistant starch (RS) (Englyst et al., 2007). RDS is starch that is converted into glucose within 20 min of digestion in the small intestine while SDS takes 20–120 min to be fully digested in the small intestine (Englyst et al., 2007). RS is any starch that escapes digestion in the small intestine and proceed to the large intestine undigested (Englyst et al., 2007).

Starch digestion starts in the mouth by the salivary α-amylase which breaks starch into maltose, maltotriose, and dextrins (Butterworth et al., 2011; Brownlee et al., 2018). Digestion continues in the small intestine by secretion of pancreatic α-amylase that hydrolyzes α-1,4 linkages of dextrins into the disaccharide maltose while maltase and α-dextrinase, brush boarder enzymes, form glucose from maltose and limit dextrins (Butterworth et al., 2011)**.** Starch digestion is completed in the small intestine where the two brush border enzymes isomaltase debranches 1,6-α-bonds of limit dextrin to produce glucose while amyloglucosidase (also called glucoamylase) cleaves 1,4-α-glycosidic bonds of disaccharides to produce glucose which is transported through the enterocyte in to the blood stream (Kumar and Satyanarayana, 2009) leading to increased postprandial blood glucose level (Drozdowski and Thomson, 2006; Roder et al., 2014; Zheng et al., 2012). Due to low lipophilicity of glucose, the transfer of most glucose requires specific carrier proteins through active transport mechanism (Stringer et al., 2015). The main glucose transporter proteins include sodium dependent glucose transporter 1 (SGLT1) and glucose transporter type 2 (GLUT2) which moves glucose from apical membrane to basolateral membrane of the enterocyte (Chen et al., 2016; Stumpel et al., 2001).

## 3 Potential use of polyphenols to control glucose digestion and transport

Carbohydrates interactions with phenolic compounds is among the interactions that have generated great interest in the recent past. Phenolic compounds affect starch digestibility by inhibiting the two primary starch digesting enzymes; α-amylase and α-glucosidase (Nyambe-Silavwe et al., 2015; Sun and Miao, 2019; Miao et al., 2014) (Table 1). Hypothetically, inhibition of starch digesting enzymes can reduce the amount of starch digested in the gut and further reduce glucose released in the bloodstream (Amoako and Awika, 2016a, 2016b; Barrett et al., 2018).

**Table 1:** *In vivo* and *in vitro* Studies showing that polyphenols inhibit glucose digestion and transport.

Animal/Human Study	Design, duration, no. of animals, doses	Outcomes	References
*In vitro*	0.5 mg/mL Hog pancreatic α-amylase, 500 μl of 1% starch incubated at 25 °C for 10 min Gallic acid treatments: S1 = 100% acarbose (25μM); S2 = 100% gallic acid (25μM); S3 = 50% acarbose +50% gallic acid; S4 = 75% acarbose +25% gallic acid; and S5 = 25% acarbose +75% gallic acid	S4 had the highest inhibitory effect (80%, p < 0.05)	Oboh et al. (2015)
	100 μL of α-glucosidase solution incubated at 25 °C for 10 min Gallic acid treatments: S1 = 100% acarbose (25μM); S2 = 100% gallic acid (25μM); S3 = 50% acarbose +50% gallic acid; S4 = 75% acarbose +25% gallic acid; and S5 = 25% acarbose +75% gallic acid	S3 had the highest inhibitory effect (65.7%) which is statistically similar to S1 but different from S2, S4 and S5 (p < 0.05)	Oboh et al. (2015)
	500-μl assay volume consisted of 200 μl of amylose or amylopectin, 50 μl of PBS and 50 μl of the inhibitor (extracts from green tea, strawberry, black currant and blackberry) 200 μl of 1·25 U/ml human salivary α-amylase added and incubated for 10 min of incubation at 37 °C	Green tea inhibited maltase, sucrose and iso-maltase, IC_50_ values of 0.02, 2.3 and 2.0 mg solid/ml water, respectively Green tea, blackberry, blackcurrant and strawberry inhibited salivary α-amylase IC_50_ values = 0·009, 1·2, 1·5 and 2·5mg dry powder/ml water (amylose as substrate); 0·025, 1.6, 1.7 and 3.9 mg/ml (amylopectin as substrate)	Nyambe-Silavwe et al. (2015)
	Porcine pancreatic amylase (PPA) solution (10 mL, 280 U/mL) and amyloglucosidase (AMG) solution (1 mL, 2500 U/mL) added to 100 mg of ball milled potato starch suspended in sodium acetate buffer. Mixture incubated for 120 min at 37 °C with agitation.	As tea polyphenol/Native potato starch ratios increased from 1/50 to 1/10 (w/w), levels of slowly digestible starch gradually decreased from 80.17 to 54.36% while levels of Resistant starch increased from 16.97 to 36.53% (p < 0.05)	Lv et al. (2019)
Human study	16 healthy volunteers fed on polyphenol and fibre rich foods (PFRF). PFRF was administered and blood samples collected at 0 (fasted), 15, 30, 45, 60, 90, 120, 150 and 180 min after consumption	Statistically significant (p < 0.01), dose dependent decrease in the mean postprandial glucose (-27.4 for low dose and -46.9 for high dose) Reduction of insulin area under curve (AUC) for PFRF meal (-46.9).	Nyambe-Silavwe et al. (2015)
Mice study	After 16 h overnight fast, mice in different groups were fed different rations of tea polyphenols (TPs) and native potato starch (NPS): TPs/NPS = 1/25, 1/10 w/w and starch at 1/kg body weight Blood samples collected from lateral tail vein at 0, 15, 30, 45, 60, 90 and 120 min after gavages	Native potato starch (control) had blood glucose peak after 30 min (4.9–5.6 mmol/L) while TPS/NPS combinations reached glycemic peak 45 min after gavages, showing the potential of polyphenols in delaying the glycemic peak	Lv et al. (2019)
	Male mice placed into five groups (n = 8), and were given young apple polyphenols (YAP) (150 mg/kg b.w.,), phlorizin (150 mg/kg b.w., i.g.), chlorogenic acid (150 mg/kg b.w., i.g.), tannic acid (150 mg/kg b.w., i.g.) or saline (control) for 6 days. 7^th^ day: All mice treated with starch (5 g/kg b.w,. Blood glucose measured at 0, 30, 60, 90, 120 min after feeding.	YAP decreased the peak blood glucose level by 13.3% at 60 min and peak insulin level by 16.2% at 90 min compared to the control (p < 0.05). Decreasing effects of the phenolic compounds ordered as: Tannic acid > phlorizin > YAP > chlorogenic Acid.	Li et al., 2019
*In vitro*	Wheat bread and gluten-free bread were co-digested *in vitro* with different amount of tea polyphenols [0% GTE) 1% GTE, (50 mg) 2.5% GTE, (125mg) 5% GTE, (250 mg), 10% GTE, (500 mg), 20% GTE, (1000 mg)	Percentage of digested starch at each GTE levels (%) were 87.0, 84.0, 79.2, 64.5, 53.8 and 23.3 for 0% GTE, 1% GTE, 2.5% GTE, 5% GTE, 10% GTE and 20% GTE, respectively.	Kan et al., 2020a, Kan et al., 2020b
*In vitro* study	Addition of green tea extract (GTE) at 0.45%, 1%, and 2% concentration levels significantly reduced the glycaemic potential of baked and steamed bread	Bread with 2% GTE had significantly lower levels at 90 min.	
Mice model	Mice were given common corn starch (5 g/kg b.w), glucose (2 g/kg b.w., i.g.), maltose (2 g/kg b.w., i.g.), or sucrose (2 g/kg b.w., i.g.) alone or in combination with EGCG (100 mg/kg b.w)	Co-treatment with EGCG significantly reduced postprandial blood glucose levels after administration of common corn starch compared to control mice (50 and 20% reduction in peak blood glucose levels and blood glucose varea under the curve, respectively). EGCG had no effect on postprandial blood glucose following administration of maltose or glucose.	Forester et al. (2012)

Evidence has shown that pure polyphenols and phenolic extracts from different sources have inhibitory activities against α-amylase and α-glucosidase (Sun and Miao, 2019; Yilmazer-Musa et al., 2012; Sun et al., 2016). Through *in vitro* studies, diverse sources of phenolics such as gallic acid, tea phenolic extracts and blue maize anthocyanins have also been reported to slow starch digestion (Guzar et al., 2012; Camelo-mendez et al., 2016; Lv et al., 2019; Peng et al., 2016). In a study on the effect of Mexican blue maize anthocyanins on starch digestibility, Camelo-Mendez et al. (2016) tested different levels of maize anthocyanins and their effects on pancreatic α-amylase and amylo-glucosidase enzymes. Addition of blue maize extracts up to 75% of starch by weight led to modification of the *in vitro* starch fractions such that RDS decreased by 1.2-fold while RS increased by 2.0 times suggesting reduced starch digestion (Camelo-Mendez et al., 2016). Rocchetti et al. (2018) corroborate these findings by reporting increase in RS from pigmented maize flours after *in vitro* starch digestion.

**Figure 1:**
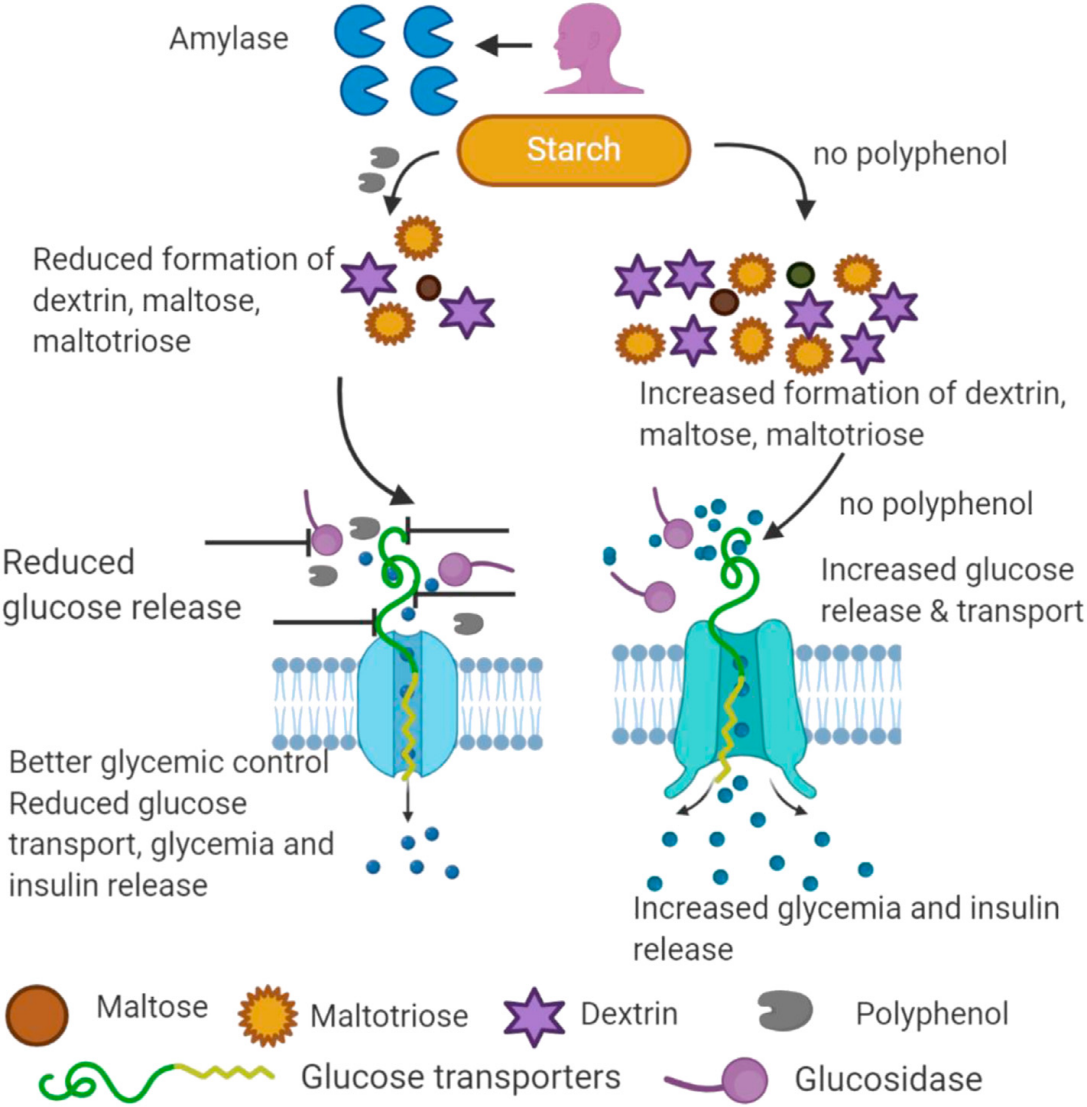
Plausible mechanisms by which polyphenols inhibit starch digesting enzymes and reduces glucose transport across the epithelia. The left side shows that polyphenols can inhibit amylases leading to reduced formation of maltose, maltotriose, and dextrins. Further, polyphenols also reduce formation and transport of glucose in the brush border membrane by inhibiting glucosidases and glucose transporters. This leads to reduced glycemia and insulin secretion. The right side of the figure shows that when there is no inhibition, there is increased digestion and transport of starch leading to increased glycemia and insulin secretion.

In other studies, Hogan et al. (2010) and Zhang et al. (2010) found that both phenolic extracts and polyphenols retarded starch digestion through inhibition of alpha glucosidases. Additionally, polyphenols inhibited *in vitro* glucose transporters thereby slowing glucose uptake by Caco-2 intestinal cell lines (Muller et al., 2018; Villa-Rodriguez et al., 2018; Pico et al., 2019; Chung et al., 2019). Dietary polyphenols have been reported to inhibit expression of SGLT1 and GLUT2 at the brush border membrane suggesting that this could slow uptake of glucose into the blood stream (Figure 1) **(**Barberis et al., 2016; Kwon et al., 2007).

The effects of dietary polyphenols go post digestion and transport of simple sugars within the gut. In cellular systems, dietary polyphenols have been reported to increase glucose uptake by cells in different tissues of the body. For example, Breen et al. (2008) and Xiong et al. (2018) found out that resveratrol and ergosterol, respectively, increased glucose transport into muscle cells through stimulating glucose transporter 4 (GLUT4) expression. Additionally, Teng et al. (2017) reported that protopanaxadiol enhanced the glucose transport into liver cells (HepG2 cells). Similarly, tea polyphenols at a dosage of 1/10 (w/w) of tea polyphenol/starch had been shown to activate insulin receptors and glucose absorption in the insulin-sensitive tissues (Lv et al., 2019). The stimulation of glucose transport into these tissue cells can also help reduce the blood sugar level. Some studies highlighting the impacts of polyphenols on starch digestion are summarized in Table 1.

From literature, there is proof that molecular weight of polyphenols has a direct effect on the inhibition capacity with monomers of low molecular weight showing lesser inhibition than polymeric polyphenols such as tannins (Amoako and Awika, 2016a; Zhu, 2015). The structure of the tannins are more hydrophobic due to abundance of hydroxyl groups which strengthen the interactions with carbohydrates or enzymes through hydrogen bonding (Table 2) (Sun and Miao, 2019; Amoako and Awika, 2016a, 2016b; Liu et al., 2017). The degree of polymerization was observed to affect enzyme inhibition activity of polyphenols as concluded by a study on red rice proanthocyanidins (Liu et al., 2017). In this study, there was higher inhibition of enzymes because proanthocyanidins with higher degree of polymerization had more free hydroxyl groups leading to stronger bonds with the active sites of pancreatic amylase. On the contrary, it was hypothesized that proanthocyanidins with lower degree of polymerization formed weaker bonds with enzymes due to the lower number of hydroxyl groups, implying that some active sites of the enzyme were uncompetitively occupied (Liu et al., 2017). Sorghum proanthocyanidins can potentially bind with amylose and linear fragment of amylopectin through hydrophobic force, reducing the digestibility of high amylose starch (Barros et al., 2014). This is supported by Bordenave et al. (2014) who explained in their review that plant bioactives can form bonds with macronutrients majorly through van der Waals bonds and such interactions are governed by bioactives' degree of polymerization, presence of galloyl groups and exterior hydroxyl groups (Table 2). Some authors have also proposed that anthocyanins compete with starch for the enzymes thereby lowering digestion (Sui, Zhang and Zhou, 2016b). Alternatively, crosslinks can occur between anthocyanins and starch thereby hindering amylolytic breakdown by enzymes in the intestines (Zhu, 2015). From product development perspective, it is important to note that addition of phenolic compounds has been shown to reduce the final viscosity of starches (Wu et al., 2011, 2015) as hydroxyl groups compete with starch for water and ultimately reducing viscosity.

**Table 2:** Some proposed mechanisms by which plant bioactives slow starch digestion.

Bioactives	Mechanism of Action	References
Tannins	Starch-tannin complex formed through hydrogen and hydrophobic interactions makes the starch inaccessible to the enzymes	Sun and Miao (2019) Amoako and Awika, 2016a, Amoako and Awika, 2016b
Proanthocyanidins with high degree of polymerization	Strong hydrogen bonding with the enzyme at the active site, causing competitive inhibition Aromatic rings of proanthocyanidins alters the microenvironment leading to inhibition. Interaction between amylose/amylopectin-proanthocyanidins through hydrophobic force	Liu et al. (2017) Sun et al. (2018)
Proanthocyanidins with lower degree of polymerization	Forms weaker hydrogen bonds with the enzyme, some active sites are uncompetitively occupied	Liu et al. (2017)
Anthocyanin	Formation of cross-linked networks with starch preventing amylolytic attacks; alteration of starch fractions with increased resistant starch and decreased rapidly digestive starch, leading to lower starch hydrolysis index Hydrogen bonding with enzymes at active sites leading to competitive inhibition	Zhu (2015) Camelo-Mendez et al. (2016) Rocchetti et al. (2018) Sui et al. (2016b)
	Inhibit the expression of glucose transporters SGLT1 and GLUT2	Li et al. (2019)
Catechins	Hydroxyl group on the flavan ring and benzene ring of catechins interact with active sites of enzyme between glycone site -1 and aglycone site +1, forming phenol-protein complex which eliminates enzyme access to the substrate	Lv et al. (2019) Miao et al. (2014)
Tea Polyphenols (TPs)	Formation of hydrogen-bond mediated amylose-tea polyphenolscomplex leading to aggregation of amylose molecules, hence less exposure to the enzymes. Stimulation of insulin production and modification of glucose release from the liver Molecular hydroxylation at hydrogen atom positions because the hydroxyl group interact with amino acid residues at the active sites of the α-amylase enzyme.	Lv et al. (2019) Miao et al. (2014) Lo Piparo et al. (2008)

In food systems, concentration of polyphenols affects the degree of starch digestion or transport**.** Incorporation of tea polyphenols in potato starch reduced starch digestibility by increasing the proportions of RS from 17.0 to 36.5% (Lv et al., 2019) while potato polyphenols dose dependently (10–300μM) decreased digestive activity of glucosidase by 3.6–7.7% but had no effect on amylase (Moser et al., 2018). Nevertheless, polyphenols reduced expression of d7-glu transport by 4.5–83.9% suggesting glucose transport through the differentiated Caco-2 human intestinal cell monolayers was reduced. This was consistent with an earlier study that reported reduction in release of glucose from 5.9-15% which consequently reduced transport of d7-glu by 10–38% when starch meals were co-digested with grape juice polyphenols (Moser et al., 2018). Taken together, these findings suggest that concentration or type of polyphenols influence the extent of inhibition which may occur as a result of polyphenol-enzyme complexion which modifies enzyme functionality or due to inhibitory effect of polyphenols on glucose transporter thereby reducing the delivery of glucose to basolateral membrane of the brush boarder into the blood stream. Plausible mechanisms by which phenolic compounds inhibit starch digestion and transport are summarized in Table 2.

## 4 Advancement in the development of bioactive-rich novel foods

Extracts of polyphenols and other plant bioactives have shown promising results in inhibition of enzymes and slowing glucose absorption and there have been attempts to develop novel foods with these compounds. Consequently, phytochemicals are currently being included in a variety of foods such as bread, beverages or baked products (Jaeger et al., 2009; Coe and Ryan, 2016; Sui et al., 2016b; Budryn et al., 2016) to leverage the starch digestion inhibition potential of these compounds. Polyphenol extracts from plant-based foods are incorporated in functional foods to help alleviate type II diabetes though the effects are variable and are dependent on concentration and type of food. Addition of polyphenol extracts in white bread did not decrease satiety nor glycemic response in 13 human subjects in a crossover trial where participants were given white bread as a control, white bread with 0.4% extract of tea and white bread having 1.88% extract of baobab (Coe and Ryan, 2016). In another study, incorporation of 1.88% baobab extracts in baked bread reduced glucose response and RDS in a dose dependent manner during an in *vitro* experiment (Coe et al., 2013). These authors also did an *in vivo* experiment where low (18.5 g) and high (37 g) doses of baobab dissolved in water and consumed alongside bread significantly reduced glucose response at 60, 120 and 180 min compared to control.

The effect of amount or concentration of bioactive compounds incorporated in a food on degree of starch digestion inhibition was observed by Sui et al. (2016b) through an *in vitro* investigation when they noticed that increase in anthocyanin extract in bread was inversely proportional to starch digestion rates. Incorporation of anthocyanin extract at 4% led to reduced elasticity and increased density in the bread compared to the control or when only 2% of the anthocyanin extract was incorporated suggesting increased density or viscosity may partly explain the inverse relationship between anthocyanin concentration and starch digestion. Similarly, incorporating 1–5% coffee bean flour in bread reduced starch digestion up to 10% suggesting coffee bean flour was inhibitory to starch digesting enzymes (Swieca et al., 2018). These authors observed that additional phenolic compounds came from bread as digestion loosens the food matrices thereby freeing more phenolic compounds. Protein band intensities were higher in bread with higher amount of coffee powder extracts than control, indicating the formation of protein/sugar-phenolics polymeric structure through crosslinking within the food matrix which may be inaccessible by digestive enzymes thereby reducing digestion. However, in some instances, such crosslinks can also reduce the amount of phenolics that participate in enzyme inhibition, leading to reduced enzyme inhibition and increased starch digestion. Incorporation of green tea catechins (0.45–2.0%) in steamed or baked bread lowered the glycemic potential in a dose dependent manner. Significant reduction in released glucose was observed in bread with at least 2% of the green tea extracts relative to the control at 20, 60 and 90 min and retention of green tea catechins was higher in steamed (81–99%) compared to baked bread (75–90%) suggesting that high temperatures used during baking can facilitate losses through volatilization or crosslinking with other food components. Moreover, the extent of starch digestibility in the crust was lower compared to the crumb and it has been observed that starch in bread crust is not fully gelatinized compared to starch in the bread crumb (Primo-Martin et al., 2007). Confocal scanning microscopy images have shown that starch granules maintained their granular structure in the crust but in the crumb starch lost its granular structure indicating full gelatinization (Primo-Martin et al., 2007). Moreover, high Maillard reactions in bread crust can lead to the formation of resistant starches with slow digestion property in addition to limited water for full gelatinization of starch unlike in the crumb where there is enough moisture to facilitate complete starch gelatinization (Pellegrini et al., 2020). These findings suggest that besides bioactive compounds present in the food, subsequent food processing can impact starch digestibility.

Addition of apple peels in cake was shown to reduce glucose release *in vitro* when the level of apple peels was increased from 0 to 6 g (Jun et al., 2014). The control cake without apple peels had significantly higher RDS (182 mg/g) compared to cakes with 3 g (150 mg/g) and 6 g (127 mg/g) of apple peels though the SDS was not statistically significant among the treatments. Resistant starch was significantly higher in cakes with 6 g of apple peels (94 mg/g) compared to 3 g of apple peels incorporated in cakes (69 mg/g) and the control cake without apple peels (36 mg/g) p < 0.05. However, addition of polyphenol rich blueberries or raspberries to solid food rich in starch had no impact on glycemic response in human participants compared to when no blueberries or raspberries were added (Clegg et al., 2011). Nonetheless, compared to those who were consuming glucose solution as the reference meal, the berries enriched foods reduced blood glucose response suggesting that liquid foods or those with simple sugars are likely to exert increased glucose sparks than starch-based solid foods. In a randomized control trial, the effects of wheat or rye bread with different berries namely lingonberries, chokeberries, strawberries, and bilberries, on postprandial insulin responses were investigated (Torronen et al., 2013). They observed that when berries are incorporated in white or rye bread which is typically a fast digesting carbohydrate, there was reduced insulin responses, and rye bread showed greatest reduction in insulin response compared to the fast digesting control white bread. However, the effect of novel bread with berries on postprandial glucose response was modest and was typically observed between 0-30 min with significant improvement in glycemic response witnessed only with mixtures of berries and strawberries in bread.

**Figure 2:**
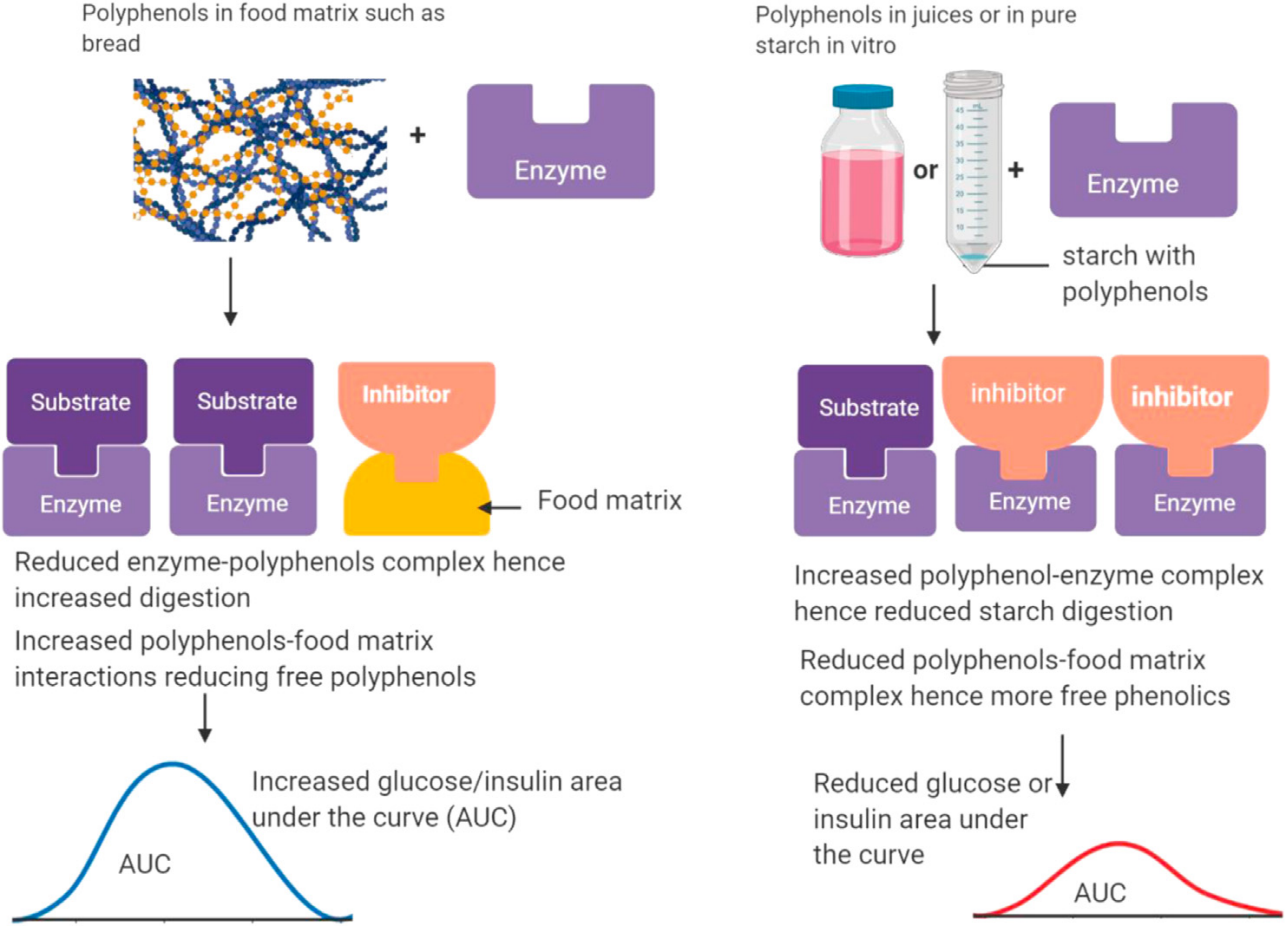
Illustration showing that phenolics in food systems may interact with food matrix reducing the amount of free phenolics available to inhibit starch digesting enzymes. The left side shows that polyphenols may interact with food matrices thus lowering enzyme inhibition and increasing digestion of starch. In the right, we postulate that in liquid foods or isolated starch, there is less interaction of polyphenols with food matrices hence more polyphenols are free to interact with enzymes leading to reduced digestion of starch.

In another randomized crossover trial, 21 healthy women participants were divided into two groups; one group consumed 150 g of lingonberries or blackcurrant purees while the second took 300 ml of lingonberry or blackcurrant nectars, each having 35 g of sucrose incorporated (Torronen et al., 2012). The reference meal used in the study was 35 g of sucrose dissolved in water. They found that intakes of sucrose with whole berries and nectars lowered insulin and glucose sparks particularly during the initial 30 min consequently leading to enhanced glycemic control. Recently, Kan et al. (2020a) observed greater starch digestion inhibition when berry extracts were co-digested with bread compared to bread fortified with berry extracts during *in vitro* assay. This reduction in inhibition in food systems is probably a consequence of polyphenols interaction with other constituents in the matrix (Bordenave et al., 2014; Kan et al., 2020a). Moreover, some of the phenolics could volatilize or degrade at high temperatures during processing of foods (Kamau et al., 2020) as baking reduced the amount of polyphenols in bread (Kan et al., 2020a). Still, some of the products of thermal degradation can inhibit starch digesting enzymes. On the other hand, thermal processing such as baking can also increase free phenolics by releasing those previously esterified to cell wall components or through thermal degradation of polyphenolic compounds (Cheng et al., 2006; Abdel-Aal and Rabalski, 2013). Generally, positive inhibition of pure starch digestion using pure polyphenols, or their extracts have been experienced *in vitro* (Yilmazer-Musa et al., 2012; Lim et al., 2019; Boath et al., 2012). These studies suggest that berries, pure polyphenols or their extracts can typically be used to inhibit starch digestion hence control postprandial glucose sparks particularly in liquid foods but their effects on starch-based foods such as bread may be reduced (Figure 2).

The protective effects against increased glycemia of phytochemical compounds found in juices have been observed in one human study where apple juice or water each containing 25 g of glucose was consumed (Johnston et al., 2002). The amounts of phloridzin were 5.7–11.9 μg/ml in clear juices and 4.1–27.3 μg/ml in cloudy juices while the chlorogenic acids contents were 35–69 and 79–256 μg/ml in the clear and cloudy juices, respectively. Individual treatments didn't impact blood glucose amount but the interaction of time × treatment was significant after 3 h showing that all the apple juices significantly reduced absorption of glucose compared to the control. Further, compared to the control, the clear juice significantly reduced plasma glucose level at 15 and 30 min, while the cloudy juice similarly reduced glucose concentration at 15, 45 and 60 minutes relative to the control. These findings suggest that commercial juices with phenolic compounds can be helpful in controlling postprandial sugar levels particularly from liquid foods which tend to have accelerated absorption of sugars compared to solid starch-based foods.

Ingestion of 150 g of bilberries puree containing 35 g of sucrose with 800 mg of polyphenols predominantly anthocyanins, flavonols, proanthocyanidins and phenolic acids significantly reduced sucrose digestion and absorption from gastrointestinal tract compared to a control (without berries) (Torronen et al., 2010). Various studies have also demonstrated that consumption of coffee, tea, wine alone or in combination with other foods significantly reduced postprandial blood glucose through delayed intestinal absorption of blood glucose (Hanhineva et al., 2010; Williamson, 2013; Lochocka et al., 2015) suggesting altered glucose transportation through epithelial cells of the gastrointestinal tract.

Some cereal foods such as pasta are fortified with polyphenols to enhance their health-giving properties such as slowing down starch digestion due to inhibition of α-amylase and α-glucosidase. Anthocyanins from grapes, berry juice concentrate and red sorghum flour were added to pasta to increase their antioxidant capacity (Khan et al., 2013; Sant’Anna et al., 2014; Sun-Waterhouse et al., 2013). Although the effect of polyphenols on starch digestibility was not specifically investigated in these studies, we now know that the health benefit provided by these foods may be partly explained by polyphenol-carbohydrate interactions that reduce starch digestion and consequent postprandial blood glucose. Co-consumption of wheat bread and gluten free bread with different amounts of polyphenols slowed starch digestion in a dose dependent manner which was influenced by the type of polyphenol (monomeric vs polymeric) and presence of gluten (Kan et al., 2020b). Addition of green tea extract to baked and steamed potatoes significantly reduced glycemic *in vitro* potential in a dose dependent manner. In mice, epigallocatechin gallate (EGCG) acutely reduced postprandial blood glucose level through a mechanism involving inhibition of α-amylase Forester et al. (2012) These results encourage consumption of tea, which is a common practice in many countries, together with meals as a simple strategy to moderate post feeding blood glucose levels and may be employed as a strategy to manage diabetes.

## 5 Conclusion

The increase in diabetes in developing countries is alarming as many people cannot cope with diabetes related complications and therefore an economical way to manage development of diabetes is required. This review presents strong evidence that co-consumption of starch rich food with polyphenol rich food is a viable alternative to managing postprandial blood glucose spike compared to use of drugs which carry risks of undesirable side effects. Consuming adequate whole grains, fruits and vegetables is more practical and should be promoted through nutritional education. However, the success of polyphenol intervention depends on the content and subsequent processing of food. The latter is very relevant as it could enable incorporation of polyphenolic compounds to foods commonly consumed. More research needs to be done on the amount of polyphenols and other bioactive compounds that could be added to foods without major changes in textural, organoleptic and nutritional quality as these factors may affect the acceptability of these novel foods.

## Declarations

### Author contribution statement

All authors listed have significantly contributed to the development and the writing of this article.

### Funding statement

This research did not receive any specific grant from funding agencies in the public, commercial, or not-for-profit sectors.

### Data availability statement

No data was used for the research described in the article.

### Declaration of interests statement

The authors declare no conflict of interest.

### Additional information

No additional information is available for this paper.
